# Records Needed for Orthodontic Diagnosis and Treatment Planning: A Systematic Review

**DOI:** 10.1371/journal.pone.0074186

**Published:** 2013-11-12

**Authors:** Robine J. Rischen, K. Hero Breuning, Ewald M. Bronkhorst, Anne Marie Kuijpers-Jagtman

**Affiliations:** 1 Department of Orthodontics and Craniofacial Biology, Radboud University Nijmegen Medical Centre, Nijmegen, The Netherlands; 2 Department of Preventive and Curative Dentistry, Radboud University Nijmegen Medical Centre, Nijmegen, The Netherlands; University of Toronto, Canada

## Abstract

**Background:**

Traditionally, dental models, facial and intra-oral photographs and a set of two-dimensional radiographs are used for orthodontic diagnosis and treatment planning. As evidence is lacking, the discussion is ongoing which specific records are needed for the process of making an orthodontic treatment plan.

**Objective:**

To estimate the contribution and importance of different diagnostic records for making an orthodontic diagnosis and treatment plan.

**Data sources:**

An electronic search in PubMed (1948–July 2012), EMBASE Excerpta Medica (1980–July 2012), CINAHL (1982–July 2012), Web of Science (1945–July 2012), Scopus (1996–July 2012), and Cochrane Library (1993–July 2012) was performed. Additionally, a hand search of the reference lists of included studies was performed to identify potentially eligible studies. There was no language restriction.

**Study selection:**

The patient, intervention, comparator, outcome (PICO) question formulated for this study was as follows: for patients who need orthodontic treatment (P), will the use of record set X (I) compared with record set Y (C) change the treatment plan (O)? Only primary publications were included.

**Data extraction:**

Independent extraction of data and quality assessment was performed by two observers.

**Results:**

Of the 1041 publications retrieved, 17 met the inclusion criteria. Of these, 4 studies were of high quality. Because of the limited number of high quality studies and the differences in study designs, patient characteristics, and reference standard or index test, a meta-analysis was not possible.

**Conclusion:**

Cephalograms are not routinely needed for orthodontic treatment planning in Class II malocclusions, digital models can be used to replace plaster casts, and cone-beam computed tomography radiographs can be indicated for impacted canines. Based on the findings of this review, the minimum record set required for orthodontic diagnosis and treatment planning could not be defined.

**Systematic review registration number:**

CRD42012002365

## Introduction

Orthodontic records are required for an orthodontic diagnosis and treatment plan [1], [2]. Although records are mainly used for these purposes, monitoring facial growth and development with or without orthodontic treatment also plays an important role in research and clinical audit [3]. Traditionally, dental casts, intra- and extra-oral photographs, different radiographic images, and clinical measurements are utilized for this purpose. Recent technological advancements such as digitized dental models, the use of digital dental set-ups to mimic the outcome of orthodontic treatment, and three-dimensional (3D) imaging of the face have led to alternative options for patient documentation. With the development of multi-slice computed tomography (MSCT) and lower-dose cone-beam computed tomography (CBCT), new and valuable tools became available for orthodontic diagnosis and treatment planning in selected cases [4]. Although a conventional two-dimensional (2D) set of records is still common, these new developments may lead to a more individualized selection of records to optimize orthodontic diagnosis and treatment planning.

To decide which documentation should be selected for a specific patient, the contribution of every separate record used for orthodontic diagnosis and treatment planning should be analyzed. Therefore, a systematic review was performed to estimate the contribution of different types of diagnostic records for treatment planning in regular orthodontic patients, and the importance of each diagnostic record separately.

## Methods

### Protocol and registration

To conduct this review, the PRISMA 2009 checklist was used [5], [6]. Inclusion and exclusion criteria were defined in a protocol. Prior to the start of this systematic review, the study was registered in the international prospective register of systematic reviews, “PROSPERO” (registration number CRD42012002365) [7].

### Eligibility criteria

Studies eligible for inclusion were those regarding the following: 1) patient records used for an orthodontic diagnosis and/or treatment plan; 2) at least two different types of records are compared; 3) the outcome variable of the study is change in treatment plan; and 4) patients of any age and sex. There was no language restriction.

Exclusion criteria were the following: 1) systematic reviews, (re)views, case reports, letters to editors; 2) treatment planning limited to the surgical part of treatment or for placement of dental implants; 3) patients with cleft lip and palate or other craniofacial anomalies; and 4) studies in animal models.

The patient, intervention, comparator, outcome (PICO) question formulated for this study was as follows: for patients who need orthodontic treatment (P), will the use of record set A (I) compared with record set B (C) change the treatment plan (O)?

### Information resources

An electronic search in the following databases was performed: PubMed (from 1948 to July 1, 2012), EMBASE Excerpta Medica (from 1980 to July 1, 2012), CINAHL (from 1982 to July 1, 2012), Web of Science (from 1945 to July 1, 2012), Scopus (from 1996 to July 1, 2012), and the Cochrane Library (from 1993 to July 1, 2012). In addition, a hand search of the reference lists of included studies was performed to identify potentially eligible studies.

### Search strategy

A list of search terms was developed and databases were selected with the help of a senior librarian specialized in health sciences.

The terms used in the search strategy were:

- *Orthodontics*: orthodontic*;
- *Treatment planning*: planning, patient care planning;
- *Dental models*: dental models, models, model;
- *Dental records*: dental records, records, record;
- *Three dimensional*: imaging three-dimensional, three-dimensional imaging, 3D imag*;
- *OPT*: Panoramic radiography, radiography panoramic, orthopantomogram*;
- *CBCT*: cone beam computed tomograph*, CBCT, spiral cone beam computed tomography;
- *Photos*: Radiography dental digital, dental radiography;
- *LHP*: Cephalometry.

The Pubmed search strategy is presented in Table 1. The search idiom was adapted for the different databases. Depending on each database, terms were searched in MeSH, title/abstract, keyword, or topic. The final search was performed on July 1, 2012.

**Table 1:** Pubmed search strategy.

Search strategy Pubmed
((“orthodontics”[MeSH Terms] OR orthodontic*[tiab]) AND ((planning[tiab]) OR (“Patient Care Planning”[Mesh])) AND ((“dental models”[MeSH Terms] OR models[tiab] OR model[tiab]) OR (“dental records”[MeSH Terms] OR records[tiab] OR record[tiab])) AND ((“Imaging, Three-Dimensional”[Mesh] OR Three-Dimensional Imaging[tiab] OR 3d imag*[tiab]) OR ((“Radiography, Panoramic”[Mesh] OR Panoramic Radiography[tiab]) OR (“Cone-Beam Computed Tomography”[Mesh] OR Cone-Beam Computed Tomograph*[tiab] OR CBCT[tiab])) OR (“Radiography, Dental, Digital”[Mesh] OR Dental Radiography[tiab]) OR (“Cephalometry”[Mesh] OR “Cephalometry”[tiab]) OR (orthopantomogram*[tiab]) OR (“dental models”[MeSH Terms] OR models[tiab] OR model[tiab])))

### Study selection

In the first step of the screening process, two observers (RR, HB) independently screened the retrieved records on the basis of title and abstract according to the eligibility criteria. After reviewing the title and abstract, articles were classified as included, excluded, or unclear. Any disagreements were resolved by discussion and consensus.

In the second step, the full text of articles, classified as included, excluded, or unclear were then independently screened and classified by two observers (RR, HB). Disagreements were resolved by discussion and consensus.

Finally, a hand search of the reference lists of the included studies was performed (RR, AK).

### Data extraction

One author (RR) extracted the relevant data from the included studies. From each included study, differences in orthodontic treatment proposal based on information from two different sets of records were evaluated. The second author (HB) checked the extracted data. Disagreements between the two researchers were resolved by discussion and consensus.

### Risk of bias in individual studies

To evaluate the methodological quality of the included studies, the checklist of the Quality Assessment of Diagnostic Accuracy Studies (QUADAS-2) developed by Whiting et al [8] was used (Table 2). The QUADAS checklist includes an assessment of “risk of bias” and “concerns regarding applicability”. This assessment was performed independently by two investigators (RR, HB). Disagreements between the QUADAS scores of the observers were resolved by discussion. The researchers had access to all data, authors, journals, and results of the publication.

**Table 2:** Description of the Quality Assessment of Diagnostic Accuracy Studies tool (QUADAS) −2 [8].

Domain	Patient Selection	Index Test	Reference Standard	Flow and Timing
**Signaling questions (yes, no or unclear)**	**S1** Was a consecutive or random sample of patients enrolled?	**S4** Were the index test results interpreted without knowledge of the results of the reference standard?	**S5** Is the reference standard likely to correctly classify the target condition?	**S7** Was there an appropriate interval between index test(s) and reference standard?
	**S2** Was a case-control design avoided?		**S6** Were the reference standard results interpreted without knowledge of the results of the index test	**S8** Did all patients receive a reference standard?
	**S3** Did the study avoid inappropriate exclusions?			**S9** Did all patients receive the same reference standard?
				**S10** Were all patients included in the analysis?
**Risk of bias (high, low or unclear)**	**B1** Could the selection of patients have introduced bias?	**B2** Could the conduct or interpretation of the index test have introduced bias?	**B3** Could the reference standard, its conduct, or its interpretation have introduced bias?	**B4** Could the patient flow have introduced bias?
**Concerns about applicability (high, low or unclear)**	**A1** Is there concern that the included patients do not match the review question?	**A2** Is there concern that the index test, its conduct, or its interpretation differ from the review question?	**A3** Is there concern that the target condition as defined by the reference standard does not match the review question?	

### Statistics

Cohen's kappa statistics were used to assess the interrater agreement for the process of inclusion of the publications and for the quality assessment scores of the included studies. According to Landis and Koch [9], the level of interrater agreement is almost perfect if the value of Kappa (K) is 0.81–1.00, substantial if K is 0.61–0.80, moderate if K is 0.41–0.60, fair if K is 0.21–0.40, and poor if K is <0.20.

## Results

### Study selection

**Figure 1:**
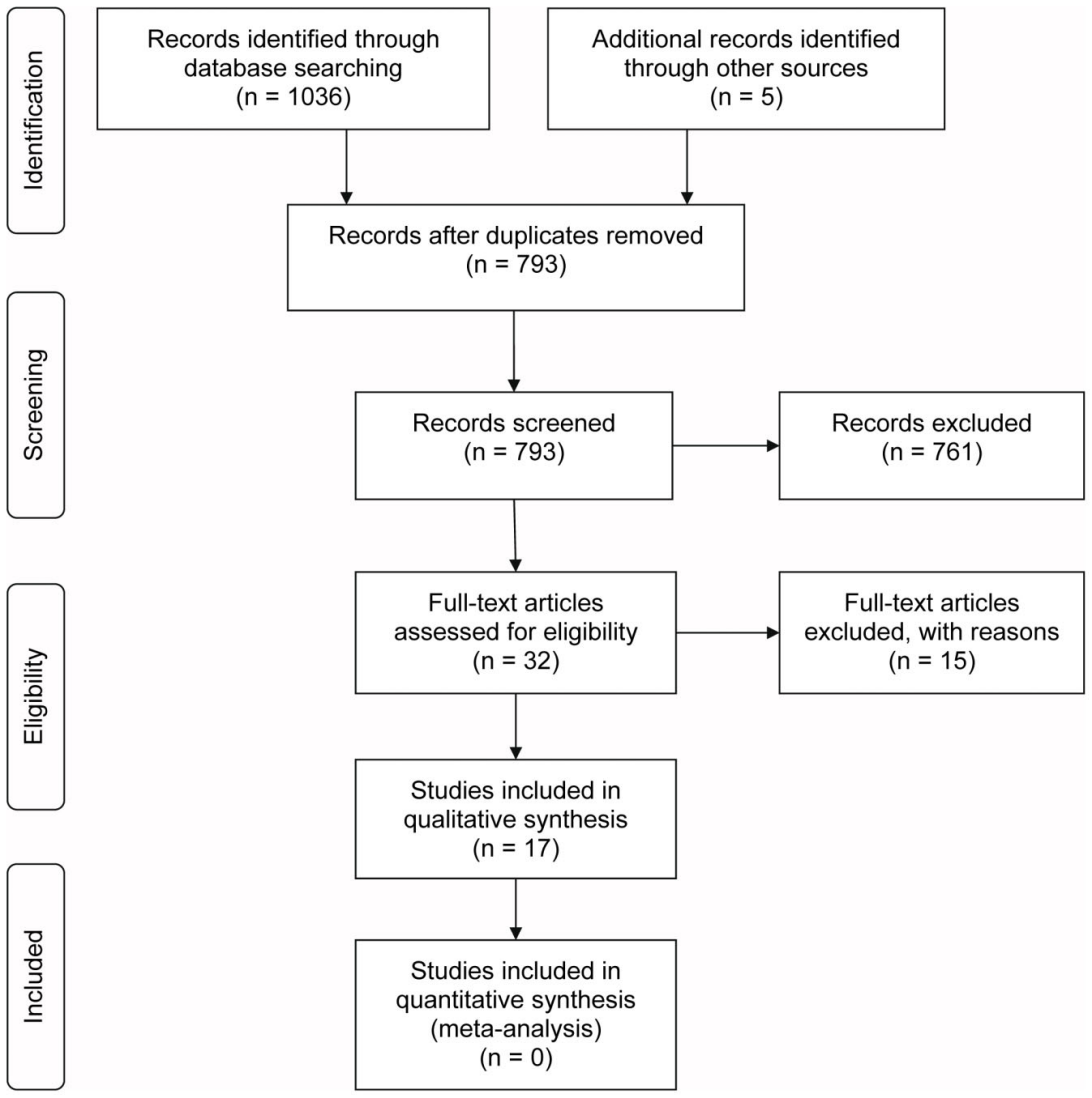
PRISMA flow diagram of study selection process.

The search of Pubmed, EMBASE Excerpta Medica, CINAHL, Web of Science, Scopus, and Cochrane Library provided a total of 1036 citations, and the hand search provided 5 citations. After adjusting for duplicates, 793 publications remained for screening of the title and abstract. Of these, 761 publications were excluded because they did not fulfill the eligibility criteria. For full text assessment of eligibility, a total of 32 studies remained. Of these, 15 studies were excluded for various reasons; 5 were excluded because the full text publication could not be retrieved, whereas 10 did not fulfill the eligibility criteria. Finally, a total of 17 studies met the inclusion criteria; 12 studies originated from the electronic databases, and 5 studies from hand searches of the references of the included studies. Figure 1 shows the PRISMA flow diagram for this study [6].

Cohen's kappa for the interrater agreement for full text assessment of eligibility was 0.606 indicating that the reliability of the process of study selection was substantial [9].

### Study characteristics

The selected studies could be divided into two groups as follows: A) studies not focused on impacted teeth, and B) studies focused on impacted teeth.

In group A (Table 3), 3 studies compared treatment plans based on a record set without radiographs versus a record set with 2D radiographs on demand [10]–[12]; 6 studies compared treatment plans based on a specific record set versus a record set including a cephalometric radiograph and analysis [13]–[18]; 2 studies compared a record set including plaster casts versus a record set including digital casts [19], [20]; and 1 study compared a record set with plaster casts mounted in an articulator versus a record set without a mounted model [21].

**Table 3:** Outcome measures of group A (studies not focused on impacted teeth).

First author	Year	Number of patients	Characteristics of patients	Number and type of examiners	Reference Standard	Index test	Occasions (interval)
**Record set versus 2D X-rays on demand**							
**Atchison KA ** [**10**]	1991	6	3× Class I and 3× Class II	39 ortho	anamnesis+dental casts+extra- and intraoral photographs	anamnesis+dental casts+extra- and intraoral photographs with extra X-ray radiography on demand	1
**Atchison KA ** [**11**]	1992	6	3× Class I and 3× Class II	39 ortho	anamnesis+dental casts+extra- and intraoral photographs	anamnesis+dental casts+extra- and intraoral photographs with extra X-ray radiography on demand	1
**Bruks A ** [**12**]	1999	70	Class I and Class II	4 pgs	anamnesis+dental casts+extra- and intraoral photographs	anamnesis+dental casts+extra- and intraoral photographs with extra X-ray radiography on demand	1
**Record set versus lateral headplate**							
**Greenhill R ** [**13**]	1979	30		11 (4 ortho; 4 dent; 1 period; 2 admini)	dental casts+LHP+tracing+colour photographs	1) dental cast; 2) dental casts+LHP+tracing	3 (4–5 weeks)
**Silling G ** [**14**]	1979	6	2× Class II/2, 2× Class I, 2× Class II/1	24 ortho	dental casts+extra oral photographs+periapical radiographs+LHP+tracing	dental casts+extra oral photographs+periapical radiographs	1
**Han UK ** [**15**]	1991	57	Class II/1	5 ortho	dental casts+extra oral photographs+OPT+LHP+tracing	1) dental casts; 2) dental casts+extra oral photo's; 3) dental casts+extra oral photo's+OPG; 4) dental casts+extra oral photo's+OPG+LHP	5 (1 month)
**Pae EK ** [**16**]	2001	80	Class I, Class II/2, Class III, open bites, bimaxillary protrusion	16 ortho	dental casts+LHP (no tracing)	dental casts	2 (minimal 1 week)
**Nijkamp PG ** [**17**]	2008	48	Class II	14 (4 ortho; 10 pgs)	dental casts+LHP+tracing	dental casts	4 (≥1 month)
**Devereux L ** [**18**]	2011	6	2× Class I, 3× Class II, 1× Class III	199–>114 ortho	anamnesis+dental casts+extra- and intraoral photographs+OPT+LHP+tracing	anamnesis+dental casts+extra- and intraoral photographs+OPT	2 (8 weeks)
**Plaster casts versus digital casts**							
**Rheude B ** [**19**]	2005	30->7 selected		7 ortho	Medical and dental history+extra- and intraoral pictures+OPT+LHP+plaster casts	Medical and dental history+extra- and intraoral pictures+OPT+LHP+digital casts	2 (maximal 30 minutes)
**Whetten JL ** [**20**]	2006	10	Class II	20 ortho	Extraoral photographs+OPT+LHP+tracing+plaster casts	Extraoral photographs+OPT+LHP+tracing+digital casts	2 (≥1 month)
**Plaster casts without articulator versus plaster casts with articulator**							
**Ellis PE ** [**21**]	2003	20		10 ortho	Extraoral photographs+OPT+LHP+tracing+plaster casts	Extraoral photographs+OPT+LHP+tracing+plaster casts articulated in the articulator	3 (minimal 2 weeks)

ortho = orthodontist(s); pgs = postgraduate(s); dent = dentist(s); period = periodontist(s); admini = administrative personnel; LHP = lateral headplate; OPT = orthopantomogram; 2D = two-dimensional.

In group B (Table 4), studies focused on treatment planning for patients with impacted teeth, 2 studies compared 2D radiographs versus MSCT [22], [23] and 3 studies compared 2D radiographs versus CBCT [24]–[26].

**Table 4:** Outcome measures of group B (studies focused on impacted teeth).

First author	Year	Number of patients	Number of canines	Number and type of examiners	Reference standard	Index test	Occasions (interval)
**2D X-ray versus MSCT**							
**Bjerklin K ** [**22**]	2006	80	113	1 ortho	Intraoral X-rays+OPT+LHP (some cases)	MSCT	2 (>10–12 months)
**Bjerklin K ** [**23**]	2008	3		157 ortho	OPT+intraoral periapical	MSCT (2 of the three cases)	?
**2D X-ray versus CBCT**							
**Haney E ** [**24**]	2010	18	25	7 (4 ortho; 3 surg)	OPT+occlusal+2 periapicals	CBCT	1
**Botticelli S ** [**25**]	2011	27	39	8 (5 pgs; 3 ortho)	OPT+LHP+periapical	CBCT	?
**Wriedt S ** [**26**]	2012	21	29	26 (10 ortho; 8 surg; 8 dent)	dental casts and OPG	dental casts and CBCT	2 (>2 weeks)

ortho = orthodontist(s); pgs = postgraduate(s); dent = dentist(s); surg = dental surgeon; LHP = lateral headplate; OPT = orthopantomogram; CT = computer tomography; CBCT = cone beam computed tomography; MSCT = multi-slice computed tomography; 2D = two-dimensional.

### Quality assessment

Cohen's kappa for the interrater agreement for all 17 criteria (Table 2; S1–S10, B1–B4, A1–A3) of the QUADAS-2 quality assessment was between 0.364 and 1. Ten items had a kappa of 0.61 or higher and were indicated as substantial or almost perfect [9]. Only B2 (Could the conduct of the index test have introduced bias?) and A2 (Is there concern that the index test, its conduct, or its interpretation differ from the review question?) had an interrater agreement below 0.41.

**Figure 2:**
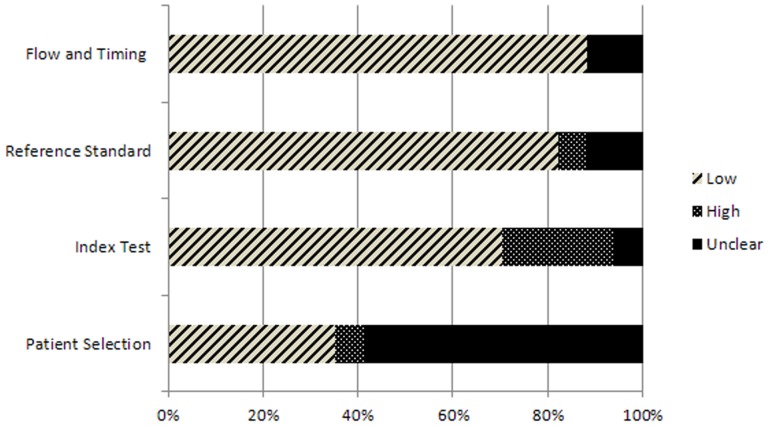
Proportion of studies with low, high, or unclear characteristics regarding ‘risk of bias.’

**Figure 3:**
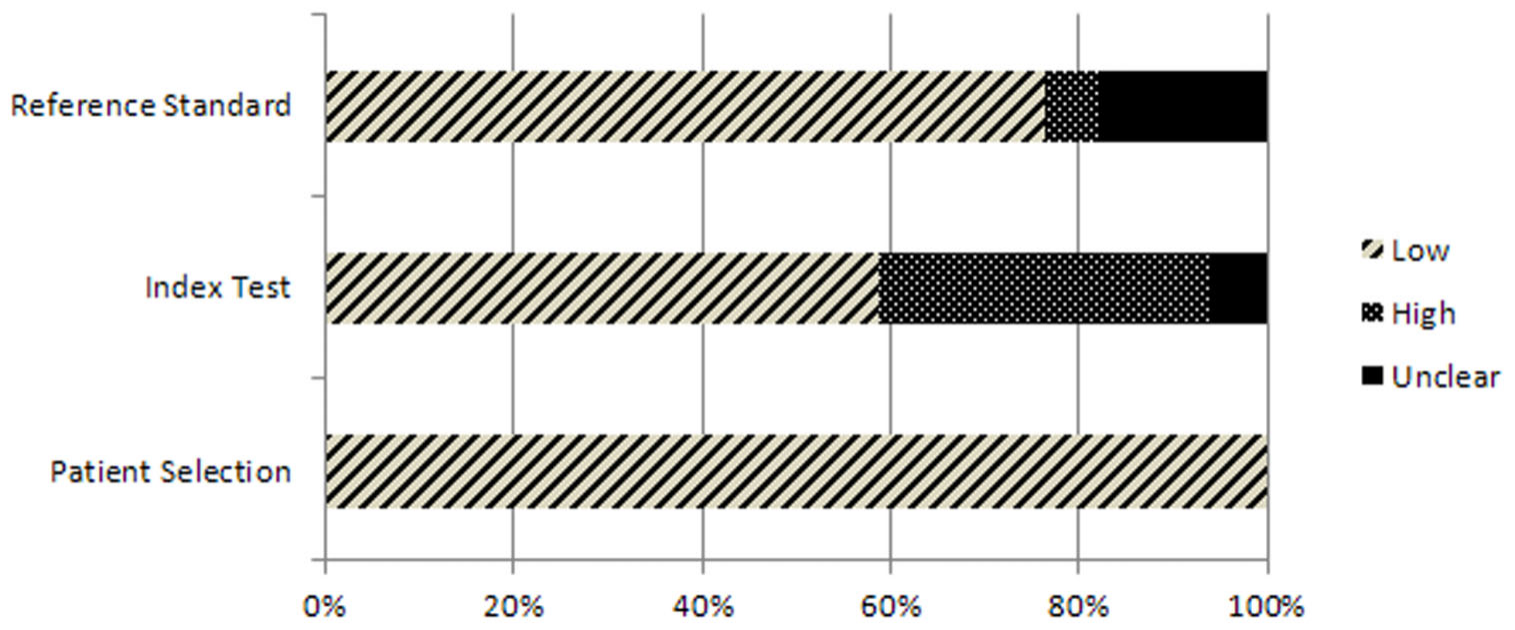
Proportion of studies with low, high, or unclear characteristics regarding ‘applicability concerns.’

Table 5 and Figures 2 and 3 show the results of the QUADAS-2 assessment. Only 4 out of 17 studies were rated as having a low risk of bias and minimal concern regarding applicability in all domains [15], [17], [20], [24] (Table 5). For the included studies, the patient selection was rated as an unclear risk of bias (Figure 2) but as having a low risk of bias regarding applicability concerns (Figure 3).

**Table 5:** Results of the quality assessment of the included studies using the QUADAS-2 instrument.

Study	RISK OF BIAS				APPLICABILITY CONCERNS		
	PATIENT SELECTION	INDEX TEST	REFERENCE STANDARD	FLOW AND TIMING	PATIENT SELECTION	INDEX TEST	REFERENCE STANDARD
Atchison 1991	?	No	Yes	Yes	Yes	No	Yes
Atchison 1992	?	No	Yes	Yes	Yes	No	Yes
Bruks 1999	Yes	No	Yes	Yes	Yes	No	Yes
Greenhill 1979	?	Yes	Yes	Yes	Yes	Yes	Yes
Silling 1979	?	Yes	Yes	Yes	Yes	No	No
Han 1991	Yes	Yes	Yes	Yes	Yes	Yes	Yes
Pae 2001	?	Yes	?	Yes	Yes	Yes	Yes
Nijkamp 2008	Yes	Yes	Yes	Yes	Yes	Yes	Yes
Devereux 2011	?	Yes	Yes	Yes	Yes	Yes	Yes
Rheude 2005	?	Yes	No	?	Yes	Yes	No
Whetten 2006	Yes	Yes	Yes	Yes	Yes	Yes	Yes
Ellis 2003	?	?	?	Yes	Yes	?	?
Bjerklin 2006	?	Yes	Yes	Yes	Yes	Yes	Yes
Bjerklin 2008	No	No	Yes	?	Yes	No	Yes
Haney 2008	Yes	Yes	Yes	Yes	Yes	Yes	Yes
Botticelli 2011	Yes	Yes	Yes	Yes	Yes	No	No
Wriedt 2012	?	Yes	Yes	Yes	Yes	Yes	Yes

Yes = Low Risk.

No = High Risk.

? = Unclear Risk.

Because of the limited number of high quality studies and the differences in study design, such as patient characteristics, reference standard or index test, a meta-analysis was not possible.

### Results of low risk of bias studies

Only 4 studies were scored as having a low risk of bias and a low concern regarding applicability (Table 5) [15], [17], [20], [24]. In their study on treatment planning, Han et al. [15] sequentially added documentation to the record set: first, study models were presented to 5 orthodontists; second, facial photographs were added to the set of records; third, a panoramic photograph (OPT) was added; fourth, a lateral cephalogram (LHP); and finally, a tracing was presented to the orthodontists to be used in planning an orthodontic treatment plan. They found that dental models only provided adequate information for treatment planning in 55% of cases. Nijkamp et al. [15] showed that the availability of a cephalometric radiograph and analysis did not influence treatment decisions in adolescents with a Class II division 1 malocclusion.

The study of Whetten et al. [20] showed that digital orthodontic study models are a valid alternative to traditional plaster study models in treatment planning for Class II malocclusion patients. Haney et al. [24] showed that in 27% of the cases the treatment plan changed after providing a CBCT. In 11% of the cases this resulted in a change in the extraction decision of the impacted canine. Therefore, they concluded that a CBCT radiograph can be considered a better choice for assessment of the location and treatment planning for impacted maxillary canines compared with 2D radiographs.

## Discussion

### Summary of evidence

A systematic review was performed to study the contribution of different diagnostic records for orthodontic treatment planning and the importance of each diagnostic record separately. We found that many studies deal with the accuracy of diagnostic records; for example, the accuracy of dental plaster casts compared with dental digital casts, but do not analyze the contribution of that specific record to treatment planning [27]. Only 17 studies were related to the latter outcome variable [10]–[26].

We used the QUADAS instrument, developed by Whiting et al [28] and adapted in 2011 to QUADAS-2 [8], to assess ‘risk of bias’ and ‘concern regarding applicability’ for the included studies. This tool was developed to assess the quality of primary diagnostic accuracy studies. QUADAS-2 does not generate a summary quality score for each article. Rating individual scores is, in general, a main problem in quality assessment scores. A good objective overall quality score is not possible, because of many different scoring items with different scales, importance, and assessment [29]. When applying the QUADAS-2 instrument, the main problem was that in most of the included articles patient selection was not very clearly described (Table 5). This stresses the importance of a clear description of the study methodology which would enable use of the study in a future systematic review.

Only 4 studies were scored as having a low risk of bias and a low concern regarding applicability (Table 5) [15], [17], [20], [24]. Han et al. [15] and Nijkamp et al. in a more recent study [17] showed that the availability of a cephalometric radiograph and analysis did not influence treatment decisions in adolescents with a Class II division 1 malocclusion. Both studies recommended that cephalometrics may be useful for other indications but they did not specify this statement. Other included studies with a lower QUADAS-2 score confirmed the findings of Han et al. and Nijkamp et al., concluding that cephalometric radiographs should not be taken routinely [13], [18]. It should be mentioned that in these studies, most of the time a specific malocclusion (e.g. Class II) was targeted. For patients with other malocclusions, the outcome would perhaps be different. Therefore, based on the results of this review it is still not possible to identify the patients who need cephalometric radiographs for orthodontic diagnosis and treatment planning.

The study of Whetten et al. [20] showed that digital orthodontic study models are a valid alternative to traditional plaster study models in treatment planning for Class II malocclusion patients. Also, the review by Fleming et al. [27] appeared to be in agreement with these results. Dental casts were used in all studies, but there is no evidence that dental casts are definitively needed for orthodontic diagnosis and treatment planning. For example, intra-oral photographs could be a good alternative to dental casts. Before conclusions can be drawn, more research about this topic is needed.

Regarding impacted canines, MSCT is more effective for treatment planning than conventional 2D radiographs such as an OPT, a cephalometric radiograph, or peri-apical radiographs [22]. The main disadvantage of using a MSCT is the high radiation dose involved. With the development of the CBCT, a lower dose alternative became available [30], [31] for orthodontic patients with impacted maxillary canines [24], [26]. Haney et al. [24] showed that a CBCT can be considered a better choice for assessment of the location and treatment planning for impacted maxillary canines compared with 2D radiographs. This was also confirmed by the study of Wriedt [26]. That study showed a change in 18% of the treatment plans between 2D and 3D radiographs, and a more accurate view of the location and identification of the teeth with 3D radiographs. Nevertheless, the effective radiation dose of CBCT is significantly higher compared with conventional 2D dental radiography. Vlijmen et al. [4] systematically reviewed indications for the use of CBCT scans in orthodontics, and concluded that there is no high-quality evidence regarding the benefits of CBCT in orthodontics. Recently, the SEDENTEXCT Project Group presented evidence-based clinical guidelines for selecting radiographs for different dental purposes [32]. They stated that the use of a CBCT may be indicated to assess an impacted tooth, including resorption of an adjacent tooth when the information cannot be obtained adequately by lower-dose conventional radiography. The smallest volume of a CBCT image should be selected because of reduced radiation dose.

New developments, such as magnetic resonance (MRI) imaging, the use of optical laser scanners for facial imaging, and intra-oral scanners for the dentition have been introduced. The influence of these new 3D imaging modalities of the face, on treatment planning, outcome of orthodontic treatment, and treatment evaluation has not yet been evaluated.

### Conclusions

Only a few high quality studies are available concerning records needed for orthodontic diagnosis and treatment planning. From the selected high quality studies, it can be concluded that for orthodontic treatment planning:

- cephalograms are not routinely needed for Class II malocclusions;
- digital models can be used to replace plaster casts;
- CBCT radiographs may be indicated for impacted canines.

Based on the findings of the present review, the minimum record set required for orthodontic diagnosis and treatment planning remains undefined.

## Supporting Information

Checklist S1 **PRISMA checklist.**
(DOCX)

Protocol S1 **Protocol for the systematic review as registered in PROSPERO (registration number: CRD42012002365).**
(PDF)
