# Supplementary material for: Records Needed for Orthodontic Diagnosis and Treatment Planning: A Systematic Review
Source: PLoS One. 2013 Nov 12;8(11):e74186. doi: 10.1371/journal.pone.0074186 (PMC3827061; doi:10.1371/journal.pone.0074186)
Supplement: Protocol S1 — Protocol for the systematic review as registered in PROSPERO (registration number: CRD42012002365). (PDF) [file pone.0074186.s002.pdf]

Records required for an optimal orthodontic diagnosis and treatment planning: a systematic review

Robine Rischen, Hero Breuning, Anne-Marie Kuijpers-Jagtman

Citation

Robine Rischen, Hero Breuning, Anne-Marie Kuijpers-Jagtman. Records required for an optimal orthodontic diagnosis and treatment planning: a systematic review. PROSPERO 2012:CRD42012002365 Available from
http://www.crd.york.ac.uk/PROSPERO/display_record.asp?ID=CRD42012002365

Review question(s)

Which radiographic images are required for orthodontic diagnosis and treatment planning?

Which intra oral images are required for orthodontic diagnosis and treatment planning?

Which extra oral images are required for orthodontic diagnosis and treatment planning?

Are dental models required for orthodontic diagnosis and treatment planning?

Searches

To identify publications, a literature search up to March 2012 will be performed in PubMed (1948-2012), EMBASE (1980-2012), Scopus (1996-2012), Web of Science (1945-2012), CINAHL (1982-2012), Cochrane Library (1983-2012) and Web of science (-2012).

A list of terms has been developed and databases were selected with the help of a senior librarian specialized in health sciences.

The terms used in the search strategy are:

1- Orthodontics: Orthodontics, orthodontic*

2- Treatment planning: planning, patient care planning

3- Dental models: dental models, models, model

4- Dental records: dental records, records, record

5- Three dimensional: Imaging Three-Dimensional, Three-Dimensional Imaging, 3D image, 3d imaged, 3d imagery, 3d images, 3d imaging

6- OPT: Panoramic radiography, radiography panoramic, orthopantomogram*

7- CBCT: Cone Beam Computed Tomography, Cone Beam Computed Tomograph*, CBCT, Spiral Cone Beam Computed Tomography

8- Photos: Radiography dental digital, dental radiography

9- LHP: Cephalometry

Depending on each database, terms were searched in MeSH, title/abstract, keyword or topic.

Types of study to be included

Primary publications in which at least two record alternatives are compared for patients in need for orthodontic treatment.

Condition or domain being studied

Diagnostic and treatment planning utility of different types of orthodontic records in patients with an orthodontic malocclusion who are at the start of treatment.

Participants/ population

Patients of all ages with an orthodontic malocclusion at the start of treatment.

Intervention(s), exposure(s)

A certain type of orthodontic record.

Comparator(s)/ control
Another type of orthodontic record or use of no record as comparison.

Outcome(s)

Primary outcomes
A list of records required for orthodontic diagnosis and treatment planning.

Secondary outcomes
Which records are not needed for orthodontic diagnosis and treatment planning.

Risk of bias (quality) assessment
A quality assessment instrument will be used.

Strategy for data synthesis
Data will be analyzed separately for records taken, study details will be pooled into tables and a descriptive summary will be listed. This will also assist in understanding which records are used nowadays for diagnosis and treatment planning for orthodontic patients.

Analysis of subgroups or subsets
None planned

Contact details for further information
Robine Rischen

Philip van Leydenlaan 25

6525 EX Nijmegen

R.rischen@dent.umcn.nl

Organisational affiliation of the review
Department of Orthodontics and Craniofacial Biology

Radboud University Nijmegen Medical Centre , Nijmegen, the Netherlands

Review team
Miss Robine Rischen,
Dr Hero Breuning,
Professor Anne-Marie Kuijpers-Jagtman,

Anticipated or actual start date
09 March 2012

Anticipated completion date
28 September 2012

Funding sources/sponsors
No external funding

Conflicts of interest
None known

Language
English

Country
Netherlands

Subject index terms status
Subject indexing assigned by CRD

Subject index terms
Dental Records; Humans; Orthodontics, Corrective

Date of registration in PROSPERO
09 May 2012

Date of publication of this revision
09 May 2012

Stage of review at time of this submission	Started	Completed

Preliminary searches	Yes	No
Piloting of the study selection process	Yes	No
Formal screening of search results against eligibility criteria	No	No
Data extraction	No	No
Risk of bias (quality) assessment	No	No
Data analysis	No	No
Prospective meta-analysis	No	No

PROSPERO
This information has been provided by the named contact for this review. CRD has accepted this information in good faith and registered the review in PROSPERO. CRD bears no responsibility or liability for the content of this registration record, any associated files or external websites.
